# Nonextensive Statistics in Nanoscopic Quantum Dots

**DOI:** 10.3390/nano16020094

**Published:** 2026-01-12

**Authors:** John A. Gil-Corrales, Alvaro L. Morales, Carlos A. Duque

**Affiliations:** 1 Facultad de Ciencias, Instituto Tecnológico Metropolitano (ITM)-Institución Universitaria, Campus Fraternidad, Calle 73 No. 76A-354 Vía al Volador, Medellín 050034, Colombia; johngil1275@correo.itm.edu.co; 2 Grupo de Materia Condensada-UdeA, Instituto de Física, Facultad de Ciencias Exactas y Naturales, Universidad de Antioquia UdeA, Cl 70 No. 52-21, Medellín 050010, Colombia; alvaro.morales@udea.edu.co

**Keywords:** nonextensive statistics, generalized entropy, Tsallis statistics, GaAs quantum dot, specific heat, electric field

## Abstract

Nanoscopic quantum dots exhibit discrete energy spectra and size- and shape-dependent thermal properties that cannot always be adequately described within the conventional Boltzmann–Gibbs statistical framework. In systems with strong confinement, finite size, and reduced symmetry, deviations from extensivity may emerge, affecting the occupation of energy levels and the resulting thermodynamic response. In this context, this work elucidates how GaAs quantum dot geometry, external electric fields, and nonextensive statistical effects jointly influence the thermal response of quantum dots with different geometries—cubic, cylindrical, ellipsoidal, and pyramidal. These energy levels are calculated by solving the Schrödinger equation under the effective mass approximation, employing the finite element method for numerical computation. These energy levels are then incorporated into an iterative numerical procedure to calculate the specific heat for different values of the nonextensivity parameter, thereby enabling exploration of both extensive (Boltzmann–Gibbs) and nonextensive regimes. The results demonstrate that the shape of the quantum dots strongly influences the energy spectrum and, consequently, the thermal properties, producing distinctive features such as Schottky-type anomalies and geometry-dependent shifts under an external electric field. In subextensive regimes, a discrete behavior in the specific heat emerges due to natural cutoffs in the accessible energy states. In contrast, in superextensive regimes, a smooth, saturation-like behavior is observed. These findings highlight the importance of geometry, external-field effects, and nonextensive statistics as complementary tools for tailoring the energy distribution and thermal response in nanoscopic quantum systems.

## 1. Introduction

GaAs quantum dots (QDs) are nanometric structures that have been extensively studied both experimentally and theoretically, to mention some recent works, in 2025, Abdelmajid Salhi et al. [1] investigated the optical and structural properties of coupled InAs/GaAs quantum dots obtained by molecular beam epitaxy (MBE) and separated by thin GaAs and GaAs_0.94_Sb_0.06_ spacer layers (SLs); in the same year, J. Filipovic et al. [2] demonstrated Autler–Townes splitting by resonant Raman scattering and absorption spectroscopy in a self-assembled InGaAs/GaAs QD doped with a single Mn atom. In another recent experimental work, A. Raymond et al. [3] studied a type-II GaAs/AlAs QD system to enable intervalley coupling between electronic states in the Γ and $X_{z}$ valleys. In the theoretical field, in 2025, R. Arraoui et al. [4] studied the response of an external electric field, the position of a shallow donor impurity, and geometric variations on the binding energy (BE) and polarizability of a system of four GaAs quantum dots. Artificial intelligence (AI) techniques have also been employed; N. Zeiri et al. [5] investigated nonlinear optical rectification (NOR) in GaAs/AlGaAs conical core/shell quantum dots by studying temperature variations, using a machine learning approach within the compact density matrix formalism.

GaAs crystallizes in the zinc-blenda structure, based on the SP^3^ bonds between atoms, as a consequence the energy structure forms a valence band and a conduction band separated by a gap of 1.42 eV. GaAs QDs have emerged as nanoscopic systems of great scientific and technological importance due to their unique optical and electronic properties derived from quantum confinement. These characteristics have driven their application in various fields and devices, ranging from lasers [6,7] and high-efficiency photodetectors [8,9] to solar cells [10,11,12,13], among other devices. In these systems, charge carriers are strongly confined in all three spatial dimensions. As a consequence, a discretization of the energy levels arises, which is crucial for the study of optical [14,15,16,17] and thermal properties [18,19,20,21,22] that differ significantly from those corresponding to macroscopic materials. In some core–shell systems based on these same materials, the thermal response has been investigated, including the effects of hydrostatic pressure [23,24], geometric effects in quantum rings (QR) [25,26]. In these types of materials, doping effects are also included in analyses of optical response [27].

The geometry associated with the confinement (shape and symmetry) of QDs leads to modifications in the density and degeneracy of the energy levels. This is a fundamental aspect when trying to understand and design quantum devices with direct applicability in photonics, electronics, and nanoscale thermal applications [28,29,30,31,32,33]. In this context, the study of the thermal response—particularly the heat capacity— provides direct information on the redistribution of energy levels and their corresponding occupation, the emergence of Schottky-type anomalies [34,35,36], and the system’s sensitivity to external perturbations such as electric fields [37,38].

Several studies have demonstrated that Boltzmann–Gibbs statistics satisfactorily describe many extensive systems. However, in nanoscopic systems such as QDs, where effective interactions, long-range correlations, or pronounced geometrical constraints may be present, it can be useful to employ generalized statistical models. One such model was introduced by Tsallis in 1988 [39] as a generalization of Boltzmann–Gibbs statistics. In this framework, an entropy-based nonextensivity parameter *q* is introduced, which modulates the relative weight of the energy levels: for q>1, weighted tails (high-energy states) are favored, while q<1 penalizes low-probability states, leading to abrupt cutoffs in the accessibility of states [40,41,42,43]. Through this formalism, it becomes possible to explore how correlations and the underlying statistical structure can modify the observable thermodynamic quantities in nanoscopic systems.

This work focuses on studying the effect of the shape of nanoscopic systems (GaAs QDs) while keeping the volume and cross-sectional area constant. Variations in the shape lead to significant changes in the energy level structure and, consequently, modify the thermal response of the system—including the heat capacity—when it is described using Tsallis nonextensive statistics. Additionally, the influence of an external electric field has been analyzed, as it can break certain symmetries and shift the energy levels, thereby modifying both the position and the nature of the thermal peaks and thresholds. To exclusively investigate the effects of geometry (the shape of the QDs), four different shapes—cube, cylinder, ellipsoid, and pyramid—are compared while maintaining the same cross-sectional area *A* and volume *V*.

The energy levels in each QD are obtained by solving the three-dimensional Schrödinger equation within the effective mass approximation, considering Dirichlet boundary conditions and employing the finite element method [44,45,46]. Using the calculated eigenvalues, an iterative numerical procedure was employed to construct the generalized probabilities and to compute the internal energy and heat capacity for different values of the nonextensivity parameter, with a variable number of levels. This approach makes it possible to study both regimes close to Boltzmann–Gibbs statistics (q=1) and nonextensive behaviors (q≠1). The results demonstrate that the QD shape significantly affects the energy spectrum and, consequently, the thermal response of the systems. This highlights the potential of using geometric shapes and external fields, together with statistical characterization, to design and tailor the thermal response of quantum nanostructures.

In nanoscale systems such as QDs, it is commonly assumed that they do not satisfy the necessary conditions for Boltzmann–Gibbs statistics (extensivity, ergodicity, or the thermodynamic limit). However, when strong confinement is considered, a finite number of accessible states and geometry-induced constraints can generate correlations or constraints that modify the statistical weights of states. In this context, the statistical framework proposed by Tsallis is appropriate and provides a valuable tool for studying deviations from standard thermodynamics. The nonextensivity parameter associated with this statistic, *q*, is not interpreted as a fundamental constant; rather, it is treated as a measure of how geometric confinement and finite-size effects can modify the occupation of energy levels, thereby influencing the thermal response of the systems under study.

The novelty of the present work lies in a systematic and unified analysis of the thermal response of the GaAs QD by simultaneously considering three modifications: geometric confinement, external electric field effects, and nonextensive statistical mechanics. Unlike previous studies that typically address these aspects separately, here we isolate pure shape effects by comparing cubic, cylindrical, ellipsoidal, and pyramidal quantum dots while keeping both the volume and the cross-sectional area constant. Furthermore, the use of Tsallis nonextensive statistics enables us to explore how deviations from Boltzmann–Gibbs behavior modify the occupation of energy levels and the resulting specific heat, providing a deeper understanding of thermal properties in nanoscopic systems beyond the standard extensive regime.

This paper is organized as follows: in Section 2, we present the theoretical framework, including the systems to be solved, the statistical model employed, and the details of the numerical calculation. Section 3 presents the results and discusses how the thermal properties can be affected by an external field and by the geometry of each QD. Finally, the conclusions of the work are presented in Section 4.

## 2. Theoretical Framework

**Figure 1:**
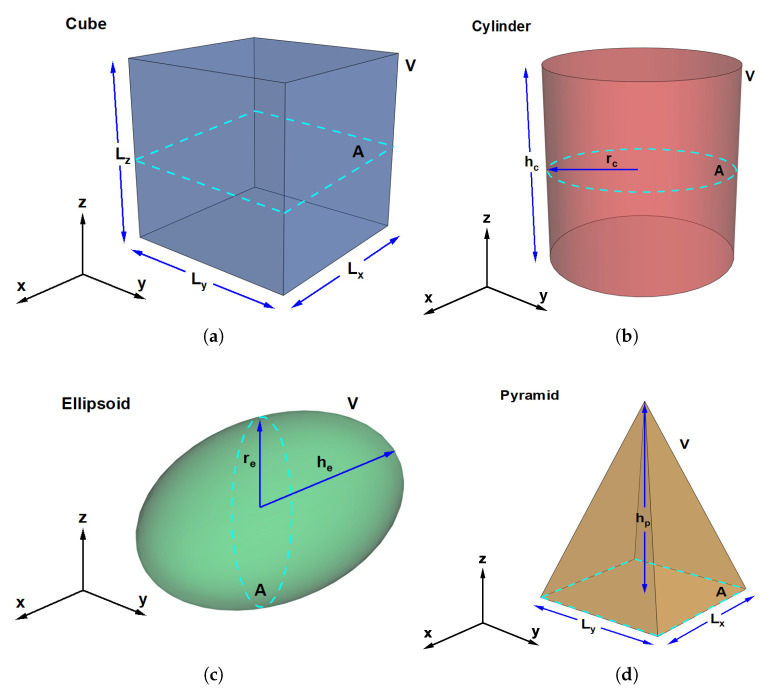
Scheme of the four QDs analyzed in this work. All QDs are characterized by having the same volume and the same cross-sectional area (see volume *V* and area *A* in each figure). These volume and area values were set based on the cubic QD with $L_{x}=L_{y}=L_{z}=10$ nm, yielding $V=10^{3}$ nm^3^ and $A=10^{2}$ nm^2^. (**a**) Cubic quantum dot. (**b**) Cylindrical quantum dot. (**c**) Ellipsoidal quantum dot. (**d**) Pyramidal quantum dot.

The system under study corresponds to a set of four GaAs QDs, as shown in Figure 1, with cubic, cylindrical, ellipsoidal, and pyramidal geometries. All quantum dots are characterized by having the same cross-sectional area *A* (represented by the dashed lines in each figure) and the same volume *V*. This condition was imposed to compare only the confinement modifications arising from the shape of the structure. The values of area and volume were fixed based on the cubic quantum dot, setting $L_{x}=L_{y}=L_{z}=10$ nm, which implies $A=10^{2}$ nm^2^ and $V=10^{3}$ nm^3^.

The characteristic size adopted in this work (with linear dimensions of approximately 10 nm) lies within the typical range reported for experimentally fabricated GaAs QDs using techniques such as molecular beam epitaxy [47,48,49]. While the absolute energy scales depend on the QD size, the qualitative trends reported here (the influence of geometry, nonextensivity, and external electric fields on the thermal response) are expected to remain valid as long as the system operates in the strong quantum confinement regime. Therefore, the conclusions drawn in this study are not limited to a particular size and reflect general features of nanoscopic QDs characterized by discrete energy spectra.

Experimental studies on QDs show that the effective size order depends on geometry, even when the dots have the same nominal volume. Researchers often compare cubes, cylinders, ellipsoid, and pyramids because their shape influences quantum confinement and optical properties. For equal volume QDs, the observed effective size order is generally: Cube > Cylinder > Ellipsoid > Pyramid. The QDs studied can be experimentally grown with associated geometric characteristics ranging from 3 nm to 15 nm commonly, by means of techniques such as: colloidal synthesis, grown epitaxially, higher confinement energy, and strongest confinement [50,51,52].

### 2.1. Numerical Calculation of the Eigenvalues

For statistical analysis, it is necessary to determine the eigenvalues associated with each quantum dot. This calculation is performed by solving the three-dimensional Schrödinger equation in the effective-mass approximation.

(1) $$\left[-\frac{\hbar^{2}}{2m^{*}}\;\nabla^{2}+V\left(\vec{r}\right)-e\;\vec{F}\cdot \vec{r}\right]\;\psi \left(\vec{r}\right)=E_{i}\;\psi \left(\vec{r}\right)\;,$$

where *ℏ* is the reduced Planck constant, $m^{*}$ is the effective mass of the electrons, *e* is the absolute value of the electron charge, $\vec{F}$ is the applied electric field, $E_{i}$ is the eigenvalue corresponding to the eigenstate $\psi (\vec{r})$, and $V(\vec{r})$ is the confinement potential given by,

(2) $$V\left(\vec{r}\right)=\left\{\begin{array}{cc} 0, & \vec{r}\in \Omega ,\\ \infty , & \vec{r}\notin \Omega \;, \end{array}\right.$$

where Ω represents the interior of each QD. To obtain the set of eigenvalues associated with each quantum dot $E_{i}$, it is necessary to solve Equation (1) including the Dirichlet boundary conditions given by $\psi (\vec{r}=\partial \Omega )=0$ where ∂Ω represents the boundary of each QD. To obtain the energy set for the different QD geometries, it is necessary to solve the effective mass Schrödinger equation using the Finite Element Method (FEM), as implemented in COMSOL Multiphysics 6.1 [53,54,55,56]. The confinement potential corresponding to each geometry is explicitly defined, and the effect of the external electric field is introduced by an additional linear potential term (Equation (1)). This approach allows for precise treatment of arbitrary geometries and boundary conditions, making it particularly suitable for the comparative analysis performed in this work.

The calculations are performed over a temperature range where thermal excitation remains comparable to the energy level separations of the QDs. At sufficiently low temperatures, only a few low-lying states contribute to the partition function, ensuring numerical stability and physical consistency. As the temperature increases, higher-energy states become populated; however, the observed saturation of the specific heat confirms that the numerical truncation of the spectrum does not affect the qualitative behavior of the results within the explored regime.

### 2.2. Nonextensive Statistics

The thermodynamic analysis is performed within the Tsallis nonextensive statistical mechanics formalism. Where the Tsallis entropy (proposed in 1988 by C. Tsallis [39,57,58,59]) is defined as:

(3) $$S_{q}=k\;\frac{1-\sum_{i=1}^{N}{\left(p_{i}\right)}^{q}}{q-1}\;,$$

where *k* is the Boltzmann constant, *N* is the total number of possible microstates of the system, $p_{i}$ is the probability of the *i*-th state, and *q* is the non-extensivity parameter that characterizes correlations in the system. In this work, the internal energy is defined using normalized *q*-expectation values, which ensures thermodynamic consistency and stability. Specifically, the internal energy is given by (internal energy constraint),

(4) $$u_{q}=\sum_{n}P_{q}\left(n,kT\right)E_{n}\;,$$

where $P_{q}\left(n,kT\right)$ are the escort probabilities defined as:

(5) $$P_{q}\left(n,kT\right)=\frac{{\left(p_{n}\right)}^{q}}{\sum_{m}{\left(p_{m}\right)}^{q}}\;.$$

This choice of constraint avoids ambiguities associated with alternative formulations of nonextensive statistics and guarantees the proper Legendre structure of thermodynamics. The term $p_{n}$ denotes standard probabilities, while $P_{q}\left(n,kT\right)$ refers exclusively to escort probabilities used in the definition of *q*-expectation values. In the case q=1, it is straightforward to show that Equation (5) reproduces the Boltzmann–Gibbs entropy $S=-k\;\sum_{i=1}^{N}p_{i}\ln\left(p_{i}\right)$, which is consistent with the usual extensive behavior of entropy:

(6) $$\lim_{q\to 1}\;S_{q}=\lim_{q\to 1}\;k\;\frac{1-\sum_{i=1}^{N}{\left(p_{i}\right)}^{q}}{q-1}\;,$$

since the total probability is given by: $\sum_{i=1}^{N}p_{i}=1$, then this limit is of the indeterminate form 0/0; therefore, applying L’Hôpital’s rule:

(7) $$S=k\;\lim_{q\to 1}\;\frac{\frac{d(1-\sum_{i=1}^{N}{\left(p_{i}\right)}^{q})}{dq}}{\frac{d(q-1)}{dq}}\;,$$

(8) $$S=\lim_{q\to 1}\;k\;\frac{-\sum_{i=1}^{N}{\left(p_{i}\right)}^{q}\;\ln\left(p_{i}\right)}{1}\;,$$

(9) $$S=-k\;\sum_{i=1}^{N}p_{i}\;\ln\left(p_{i}\right)\;.$$

For q>1, heavy tails in the distribution are favored, giving priority to less accessible states or those with long-range correlations. Finally, for q<1, low-probability states (such as high-energy excited states) are penalized, and the system exhibits a natural cut-off (with a limited number of accessible states). This is a characteristic feature of systems with strong constraints or anticorrelations.

### 2.3. Iterative Procedure

For the numerical calculation of the specific heat for each QD at arbitrary values of *q*, we start from the initial values of the probability by the stated $p_{n}^{\left(init\right)}$ and internal energy $u_{0}$ (for q=1) given by [40]:

(10) $$p_{n}^{\left(init\right)}\left(kT\right)=\frac{e^{-E_{n}/\left(kT\right)}\left(1-e^{-1/(kT)}\right)}{1-e^{-(N+1)/(kT)}}\;,$$

and

(11) $$u_{0}\left(kT\right)=\sum_{n=0}^{N}p_{n}^{\left(init\right)}\left(kT\right)E_{n}\;,$$

where *T* is the system temperature and $E_{i}$ are the eigenvalues calculated as indicated in Section 2.1. The iterative procedure employed to compute the generalized internal energy and the specific heat requires an initial probability distribution $p_{n}^{\left(init\right)}$ to start the self-consistent loop. This initial distribution is introduced solely as a numerical seed and does not carry any physical meaning. Its only role is to initialize the iteration, and it is not intended to represent an equilibrium state of the system. For the initial step in the iterative process, the functions are defined as follows.

(12) $$u_{q}\left(kT\right)=u_{0}\left(kT\right)\;,$$

and

(13) $$D_{q}\left(kT\right)=D_{0}\left(kT\right)\equiv \sum_{n=0}^{N}{\left(p_{n}^{\left(init\right)}\left(kT\right)\right)}^{q}\;.$$

From these functions that initiate the iterative process, the intermediate functions can be defined as follows:

(14) $$f_{q}\left(n,kT\right)=1-\frac{\left[(1-q)/(kT)\right]\left(E_{n}-u_{q}\left(kT\right)\right)}{D_{q}\left(kT\right)}\;,$$

(15) $$A_{q}\left(n,kT\right)={\left[f_{q}\left(n,kT\right)\right]}^{1/(1-q)}\;,$$

from these expressions, the partition function is defined as:

(16) $$Z_{q}\left(kT\right)=\sum_{n=0}^{N}A_{q}\left(n,kT\right)\;,$$

similarly, the internal energy constraint can be written as:

(17) $$P_{q}\left(n,kT\right)=\frac{A_{q}\left(n,kT\right)}{Z_{q}\left(kT\right)}\;.$$

At this point, it is possible to redefine an updated function similar to Equation (13) as:

(18) $$D_{q}^{new}\left(kT\right)=\sum_{n=0}^{N}{\left(p_{n}\left(n,kT\right)\right)}^{q}\;.$$

**Figure 2:**
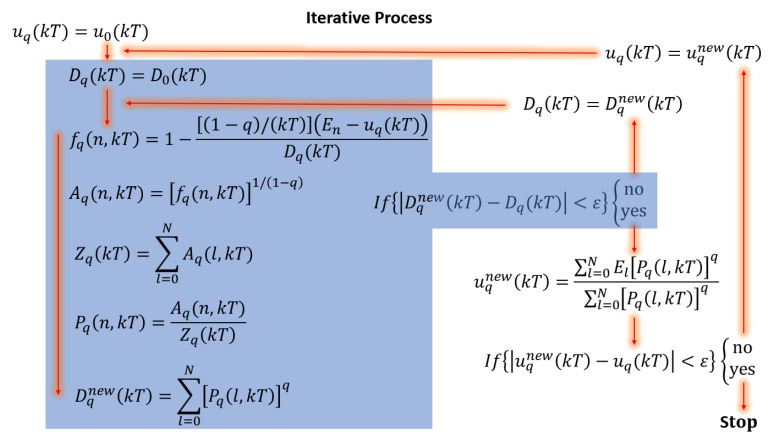
Block diagram corresponding to the iterative process, showing the equations involved in the development of the code.

By defining a tolerance parameter $\varepsilon =10^{-7}$, we can compare the convergence of the function $D_{q}$ in two successive steps as: $|D_{q}^{new}\left(kT\right)-D_{q}\left(kT\right)|<\varepsilon$, if this condition is not satisfied, the function $D_{q}\left(kT\right)=D_{q}^{new}\left(kT\right)$ is redefined. The iterative process is repeated until convergence is achieved. If the condition is satisfied, the internal energy function is actualized as

(19) $$u_{q}^{new}\left(kT\right)=\frac{\sum_{n=0}^{N}E_{n}{\left(p_{n}\left(n,kT\right)\right)}^{q}}{\sum_{n=0}^{N}{\left(p_{n}\left(n,kT\right)\right)}^{q}}\;,$$

again, the convergence of this function can be compared as: $|u_{q}^{new}\left(kT\right)-u_{q}\left(kT\right)|<\varepsilon$, as before, if this condition is not satisfied, the function $u_{q}\left(kT\right)=u_{q}^{new}\left(kT\right)$ is redefined and the iterative process is repeated until the minimum tolerance condition is met. If the condition is satisfied, the iterative process ends, and the resulting set of functions corresponds to the desired numerical solution. Figure 2 presents a block diagram summarizing the iterative process.

Once the program converges, the specific heat can finally be calculated from the internal energy using the usual expression.

(20) $$C_{q}/k=\frac{d(u_{q}\left(kT\right))}{d(kT)}\;.$$

The specific heat is computed using a central finite-difference scheme applied to the generalized internal energy (after convergence). This choice provides improved numerical stability and accuracy compared to one-sided differentiation schemes.

**Figure 3:**
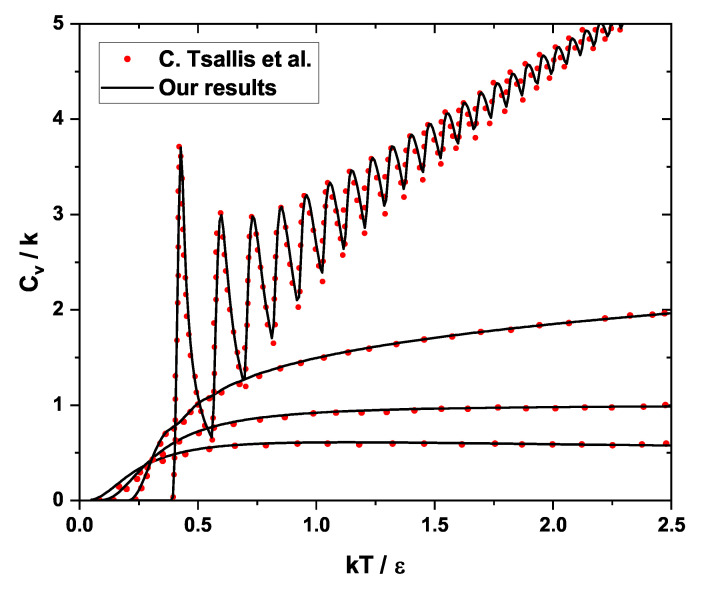
Comparison of the results obtained through our iterative process (solid black line) with the work previously reported by Tsallis et al. [40] (red dots).

To verify the validity of the described numerical method, it has been applied to the harmonic oscillator to reproduce the original work of Tsallis et al. [40], producing a significantly good comparison, as evidenced in Figure 3.

## 3. Results and Discussion

Table 1 shows the list of parameters used in this work.

**Figure 4:**
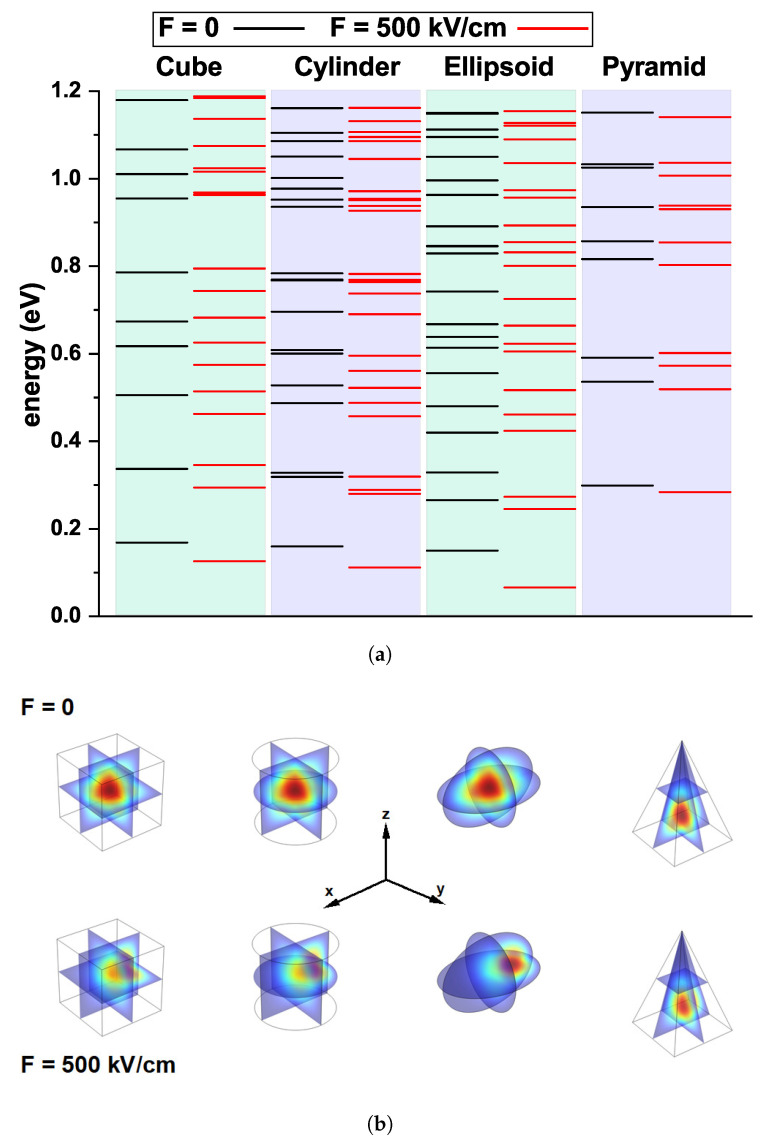
(**a**) Energy spectrum obtained by numerically solving the Schrödinger equation for each of the QDs. The black and red lines correspond to the energy levels of each QD without external fields and with an external field applied along the *x*-direction with magnitude F=500 kV/cm, respectively. (**b**) Ground-state wave function corresponding to each of the QDs. The top row shows results for F=0, while the bottom row shows results for F=500 kV/cm.

Since each quantum dot exhibits infinite confinement, there will accordingly exist an endless set of associated eigenvalues. Figure 4a shows a subset of the first eigenvalues corresponding to each quantum dot studied, obtained as previously mentioned through the numerical solution of Equation (1) using FEM. The black and red horizontal lines correspond to F=0 and F=500 kV/cm, respectively (*x*-directed electric field). From this figure, it is evident that the electric field lifts the degeneracy associated with the system’s symmetry for states aligned with the external electric field, while simultaneously inducing a redshift of the levels. According to the state ordering, the cube exhibits the most significant number of degeneracies, which are partially lifted when moving to the cylindrical geometry. However, it still retains an approximately regular character.

A different situation arises in the ellipsoidal system, where a large number of states with closer energies and a lower degree of degeneracy are observed due to its reduced symmetry. For this reason, when the electric field is applied to the ellipsoidal QD, the displacement of the states is prioritized over the breaking of degeneracies. Finally, in the pyramidal QD, which corresponds to the system with the lowest symmetry, a noticeable increase in the ground-state energy is observed compared to the other geometries. This is due to the high degree of confinement experienced by the electrons in this structure, which remain primarily near the base of the pyramid within a small region. Note that the external electric field does not significantly affect the states in the pyramid, since the probability density is highly localized and practically unaffected by the applied field along the *x* direction.

Table 2 shows the number of states for optical transitions per unit energy range, 0.4 eV. The cube, for F=0, presents the least number of states, followed by the pyramid, and the largest number for the cylinder and ellipsoid. For F=500 KV/cm, in general, there are more states in this interval; the least number is for the pyramid, followed by the cube. And the maximum for the ellipsoid and cylinder. Most of the energy transitions will lie in the infrared region (0.08–1.7 eV).

In Figure 4b, the ground state wave function for each QD is shown without the field (top row) and with the applied electric field (bottom row). With the electric field applied along the *x* direction, the electronic probability density is more significantly affected in the ellipsoidal quantum dot, since the *x* axis coincides with the major semi-axis and, therefore, the wave function exhibits a larger displacement, as evidenced in the figure. This, in turn, implies that the ground state for this geometry undergoes a more pronounced redshift, which is reflected in the corresponding spectrum. The opposite occurs in the pyramidal QD, which, as previously mentioned, is not significantly altered by the external field, as shown by the minimal displacement of the wave function associated with the application of the electric field. The energy level separations obtained for the GaAs QDs in this work are typically on the order of a few to several tens of meV (as shown in Figure 4a), which naturally places the corresponding transitions within the infrared and terahertz spectral regions (with associated energies in the approximate range of 0.41 meV to 1.24 eV). These energy scales are particularly relevant for applications involving thermal activation, low-energy optical excitations, and THz devices based on quantum-confined semiconductor nanostructures.

**Figure 5:**
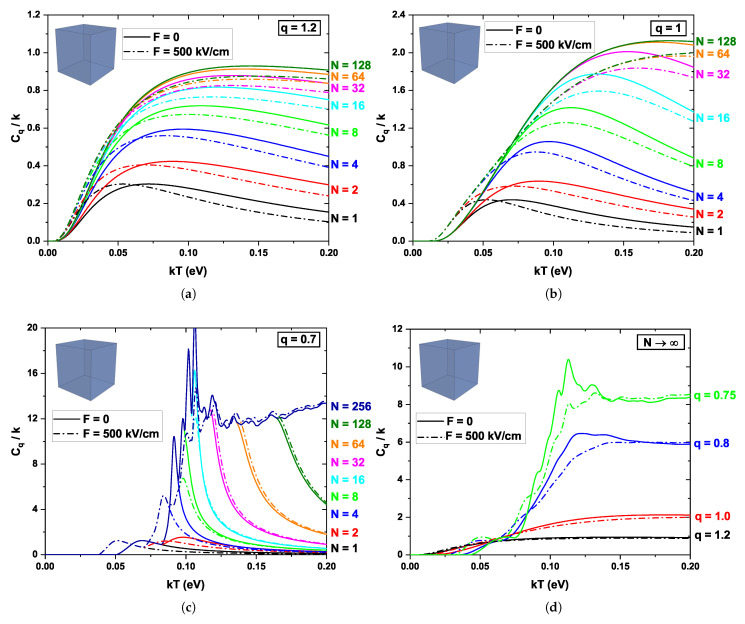
Variations of the normalized specific heat $C_{q}/k$ for the cubic quantum dot as a function of the parameter kT for different values of the non-extensivity parameter *q*. The solid and dashed lines represent F=0 and F=500 kV/cm, respectively. (**a**) $C_{q}/k$ as a function of kT for q=1.2. The colors of each line represent an increase in the number of states considered (see the *N* column on the right side of the panel). (**b**) Same as in panel (**a**) for q=1. (**c**) Same as in panel (**a**) for q=0.7. (**d**) $C_{q}/k$ as a function of kT for a very large number of states N→∞ and for different values of the parameter *q*.

Figure 5 shows the variations of the normalized specific heat $C_{q}/k$ for the cubic quantum dot as a function of the parameter kT for different values of the non-extensivity parameter *q*, calculated according to Equation (20). The solid and dashed lines represent F=0 and F=500 kV/cm, respectively.

In Figure 5a, $C_{q}/k$ is presented as a function of kT for a fixed value of q=1.2, that is, for analyzing the nonextensive behavior of the system, for different numbers of considered states (or microstates) ranging from N=1 (black curve) to N=128 (green curve). As more states are populated, the specific heat tends to increase in magnitude until it approaches a saturation limit (approximately $C_{q}/k\to 1$). This indicates that the system can absorb more thermal energy, thereby increasing its specific heat capacity. On the other hand, as the temperature rises, the value of $C_{q}/k$ increases to a maximum and then clearly decreases, a behavior that is more pronounced for smaller *N*. Since q>1, the system is superextensive; therefore, higher priority or weight is given to highly excited states in the probability distribution. Upon application of the electric field, a slight shift in the curves is observed; however, this modification is not significant because the changes in the energy spectrum are not substantial under the external field. Note that, at low temperatures, the specific heat tends to zero both with and without the external field.

On the other hand, Figure 5b shows the behavior of q=1, that is, it corresponds to the standard Boltzmann–Gibbs statistics. In this case, the peak at $C_{q}/k$ is higher than in the case q=1.2. This peak, which is more pronounced for a small number of states, can be understood by analyzing the low-temperature regime. In this case, the product kT is much smaller than the transition energy between the ground state and these states, leading to electronic accumulation in the ground state. Therefore, the internal energy practically does not change with *T*, which implies that $C_{q}\approx 0$. For the high-temperature case, the product kT is much larger than the transition energy between the ground state and these few states. This causes the states to be occupied with nearly the same probability, and since there are no additional states to be populated, a decrease in the specific heat $C_{q}\to 0$ occurs, as is observed for all curves with a small number of states (N<64) at high temperatures.

Finally, for intermediate temperatures, where the product kT is on the order of the transition energy between the ground state and these states, a slight temperature change can produce a significant change in the redistribution of the electronic population, giving rise to an apparent increase in internal energy that results in a peak in specific heat. This is a clear example of a Schottky-like anomaly that emerges in systems with a limited number of states. The electric field induces a redshift in the ground state, which in turn shifts the peak position of the specific heat to lower values of kT. In general, the action of the field results in a net shift in the specific heat curves $C_{q}/k$.

Figure 5c shows the behavior of q=0.7. This case corresponds to a subextensive regime that leads to a natural cutoff in the number of accessible states, producing abrupt, narrow peaks in $C_{q}$ whenever the temperature is sufficient to populate a new set of levels. As the number of states increases, more thresholds accumulate, and additional peaks appear in the curve. This can be understood in the same way from the probability function (Equation (17)), where we observe that $p_{q}\propto f_{q}^{1/(1-q)}$; then, $f_{q}$ becomes negative and the probability is truncated to zero. This implies that, at a certain temperature, only a few states remain accessible. On the other hand, when the temperature rises enough to include a new level, an abrupt peak in $C_{q}$ is generated. Note that such a probability cutoff does not occur in the case q>1. The action of the electric field is to change the position of the states and consequently the thresholds, thus modifying the position and height of the peaks of $C_{q}$.

Figure 5d shows the specific heat $C_{q}/k$ as a function of kT, summing over a very large number of states (numerically, N=3000 have been considered) for different values of the nonextensivity parameter *q*. Note that for q>1, the specific heat takes smaller values and is practically unaffected by the external electric field, while the behavior remains smooth. A cutoff temperature is no longer observed, and because the probability of excited states is prioritized, partial occupation of these levels already occurs at low temperatures. For the case q<1, the condition $f_{q}>0$ is required, which by Equation (14) implies that $E_{n}<u_{q}\left(kT\right)+D_{q}\left(kT\right)\left(kT\right)/\left(1-q\right)$. This indicates that there must exist a maximum accessible energy for the distribution at a given temperature. As kT increases, this maximum energy increases. When it crosses regions where the density of states is very high (for example, many degenerate levels within a short energy interval), a large number of levels suddenly begin to contribute, causing the internal energy $u_{q}\left(kT\right)$ to increase abruptly. Since the specific heat is the derivative of this function with respect to temperature, very large peaks of $C_{q}/k$ are then generated, as observed for the case q=0.8, or even more notably for q=0.75.

For q<1, the Tsallis formalism introduces an effective cutoff in the accessible energy spectrum, which leads to abrupt changes in the internal energy as a function of temperature when new states become thermally accessible. This behavior of $u_{q}$ is an intrinsic feature of the subextensive regime and is directly reflected in extremely sharp peaks in the specific heat. These features are therefore not numerical artifacts but a consequence of the generalized statistics.

Convergence with respect to the number of included energy levels is assessed using a quantitative criterion based on the stability of the specific heat. For a given geometry and external electric field, convergence is considered achieved when the maximum relative deviation, $Max|\left(C_{q}^{\left(N+1\right)}\left(kT\right)-C_{q}^{\left(N\right)}\left(kT\right)\right)/C_{q}^{\left(N\right)}\left(kT\right)|$ over the explored temperature range falls below a predefined tolerance. Once this condition is satisfied, further inclusion of higher-energy states does not produce any appreciable change in the thermodynamic quantities.

**Figure 6:**
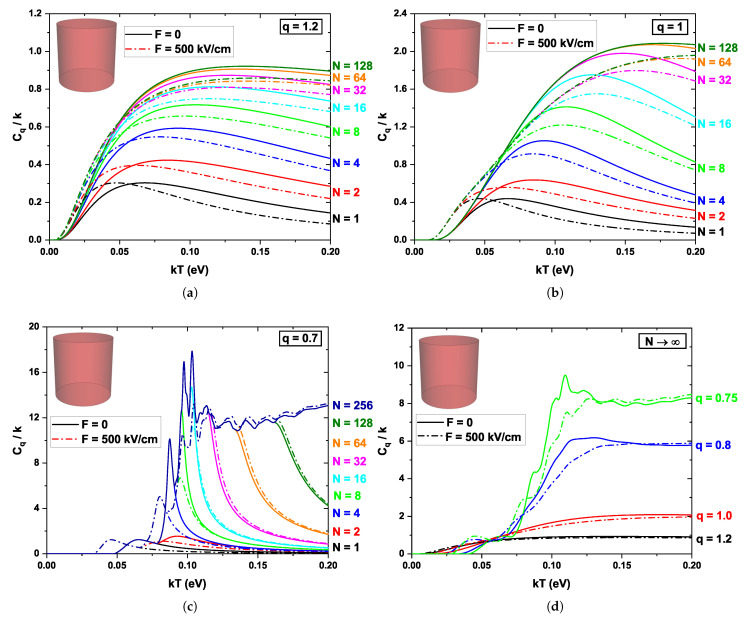
Variations of the normalized specific heat $C_{q}/k$ for the cylindrical quantum dot as a function of the parameter kT for different values of the non-extensivity parameter *q*. The solid and dashed lines represent F=0 and F=500 kV/cm, respectively. (**a**) $C_{q}/k$ as a function of kT for q=1.2. The colors of each line represent an increase in the number of states considered (see the *N* column on the right side of the panel). (**b**) Same as in panel (**a**) for q=1. (**c**) Same as in panel (**a**) for q=0.7. (**d**) $C_{q}/k$ as a function of kT for a very large number of states N→∞ and for different values of the parameter *q*.

Figure 6 shows the variations of the normalized specific heat $C_{q}/k$ of the cylindrical quantum dot as a function of kT, for different values of the non-extensivity parameter *q*, several numbers of considered energy levels *N*, and in the presence and absence of an external electric field (F=0 and F=500 kV/cm, solid and dashed lines, respectively). Panels (a)–(c) show regimes with q=1.2 (superextensive), q=1 (Boltzmann-Gibbs), and q=0.7 (subextensive), while panel (d) presents the behavior when a very large number of states is included (N→∞) for several values of *q*. The results for the cylinder are generally similar to those for the cubic case, as the energy spectra are comparable for the two QDs. For q=1 (Figure 6a), an increase in thermal excitation energy is observed, which leads to an enhancement of the specific heat as the number of levels considered increases. However, the external field induces a shift in $C_{q}/k$ without producing a significant change in the magnitude of each curve. As in cubic QD, for both q=1.2 and q=1 (Figure 6b,c), the Schottky-like anomaly effect appears when the contribution arises from a very small number of states (N<64 for q=1). For q=0.7, the behavior exhibits numerous sharp peaks, as the system’s thermal energy increases; new sets of states become populated, producing sudden jumps in internal energy and specific heat.

In Figure 6d, the specific heat $C_{q}/k$ is also shown, but considering a very large number of states for different values of the nonextensivity parameter *q*. The solid and dashed lines represent F=0 and F=500 kV/cm, respectively. For q=1.2 (solid black curve), a monotonically increasing behavior is observed at low temperatures, tending toward a saturation point at high temperatures, $C_{q}\to k$. Since excited states are favored, there is a continuous increase in the internal energy of QD with increasing thermal energy, leading to the saturation point in $C_{q}$. A similar behavior is obtained for the case q=1 (solid red curve), but the system reaches a higher saturation point, approximately $C_{q}\to 2k$. The effect of the external electric field is minimal, producing only a slight decrease in $C_{q}$. The most remarkable cases occur for q=0.8 and q=0.75 (blue and green curves, respectively), where the lowest states are favored, leading to peaks in the specific heat when the thermal energy reaches the excitation threshold. Again, the effect of the field (see dashed lines) shifts $C_{q}/k$ toward lower temperatures and also modifies the peak pattern. Note that in these cases, approximate saturation points at high temperature also appear ($C_{q}\to 6k$ and $C_{q}\to 8k$ for q=0.8 and q=0.75, respectively).

**Figure 7:**
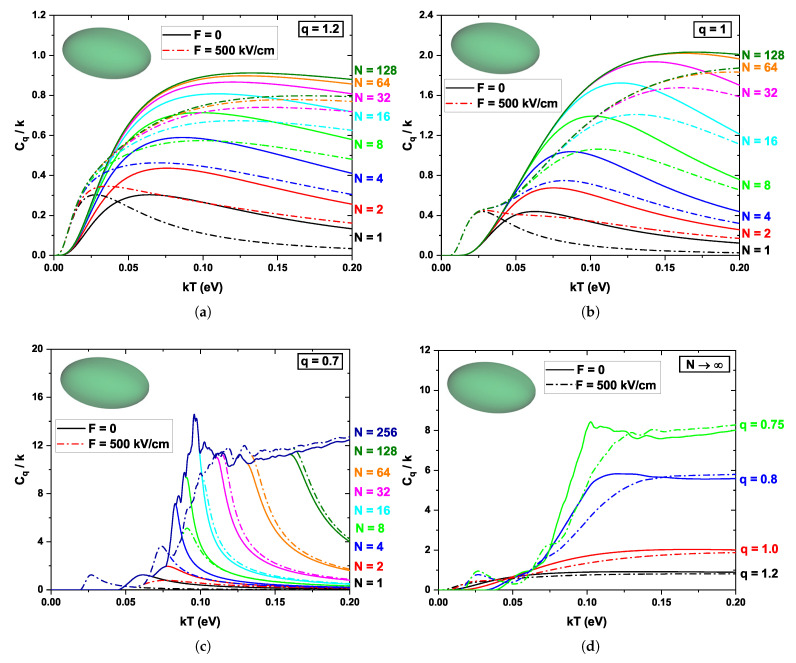
Variations of the normalized specific heat $C_{q}/k$ for the ellipsoidal quantum dot as a function of the parameter kT for different values of the non-extensivity parameter *q*. The solid and dashed lines represent F=0 and F=500 kV/cm, respectively. (**a**) $C_{q}/k$ as a function of kT for q=1.2. The colors of each line represent an increase in the number of states considered (see the *N* column on the right side of the panel). (**b**) Same as in panel (**a**) for q=1. (**c**) Same as in panel (**a**) for q=0.7. (**d**) $C_{q}/k$ as a function of kT for a very large number of states N→∞ and for different values of the parameter *q*.

Figure 7 shows the dependence of the normalized specific heat $C_{q}/k$ of the ellipsoidal quantum dot on kT, considering different values of the non-extensivity parameter *q*, several numbers of included energy levels *N*, again, in the presence and absence of an external electric field (F=0 and F=500 kV/cm, represented by solid and dashed curves, respectively). Panels (a)–(c) correspond to the cases q=1.2, q=1, and q=0.7. In contrast, panel (d) represents the behavior obtained when a very large number of states are taken into account (N→∞) for several values of *q*. In Figure 7a, a behavior similar to that previously reported for the cube and the cylinder is observed for the case F=0. When an external electric field is applied (F=500 kV/cm), the shift of the specific heat curves towards lower temperatures becomes more evident. The same occurs with the local maximum (Schottky-like anomaly), which appears for N=1 up to approximately N=16. This can be explained by referring to Figure 4a, where a more pronounced shift of the ground state towards lower energies is observed in the case of the ellipsoid when the external field is applied, which implies that less thermal excitation energy is required to populate the levels.

In Figure 7b, the behavior of the specific heat $C_{q}/k$ is shown for the case of Boltzmann–Gibbs statistics q=1 (extensive). In the absence of an applied field (F=0), a well-defined maximum (Schottky-type anomaly) appears when the number of considered states is small (N≲32). This maximum occurs in the intermediate temperature regime (kT around 0.08), where thermal energy is comparable to the energy difference between the ground state and the first excited state. Within this range, slight variations in temperature lead to a significant redistribution of the electronic population, producing a sharp increase in internal energy and, consequently, a peak in specific heat. When the effect of the external electric field (F=500 kV/cm) is included, the curves are again shifted toward lower kT values, indicating that less thermal excitation energy is required to populate higher states. As explained earlier, this is because, in the ellipsoid, the external electric field produces a more pronounced shift of the ground state to lower energies compared to the other QDs. Note that for 2<N<32, two peaks emerge in the specific heat when the external field is included, rather than a single peak as in the previous systems. In the case of the ellipsoidal QD, the presence of two local maxima in the specific heat is observed when only a few states are considered. The first peak corresponds to the excitation from the ground state to the first level, while the second peak is associated with the excitation to the second state. In the ellipsoid, the second level lies at relatively low energies (compared to the cube or the cylinder); the thermal energy required to populate it becomes accessible within the analyzed range, allowing the second peak to emerge in the curve. On the other hand, for different geometries, the second excited levels are located at higher energies; therefore, the resonance condition is not satisfied within the studied interval, preventing the appearance of the second maximum.

In Figure 7c, the case q=0.7 (subextensive) is analyzed, where priority is given to the occupation of lower states. This priority can be seen mathematically in the function $f_{q}\left(n,kT\right)$ defined in Equation (8), which, when higher states are included, may eventually take negative values. Such values are forbidden in the calculation of probability, and therefore a cut-off or truncation is imposed on the corresponding states. This implies that not all states can contribute over the entire temperature range; as thermal energy increases, states are populated abruptly, resulting in a sudden rise in specific heat capacity. Consequently, a ’discrete’ behavior of the specific heat is observed. This effect arises in both the absence and the presence of an external electric field. The action of the field produces a redshift when only a few states are considered (N<8) and a blueshift when a larger number of states are included (N>8). This can be explained by noting that, when only a small number of states are considered, the dominant contribution to the specific heat arises from first excitations of the ground state, which are strongly affected by the external field, producing a clear shift toward lower energies. On the other hand, when a larger number of states are considered, these are barely influenced by the action of the field, and some may even shift to higher energies relative to the ground state, leading to a blueshift in $C_{q}/k$.

Figure 7d shows the normalized specific heat, $C_{q}/k$, as a function of kT, considering a very large number of states, for different values of the no-extensivity parameter *q*, both with and without an external electric field (dashed and solid curves, respectively). For values of q>1, a smooth and saturating behavior is observed. This also occurs for q=1, but with a higher saturation value. This can be understood because, in this regime, the system gives greater weight to higher-energy states, leading to saturation of the internal energy. When thermal energy is supplied to the system, there are practically no significant changes in the population of the levels. For q<1, the lower-energy states become more relevant, and the level separation induces abrupt changes as thermal energy is added to the system. Note the peak in the specific heat that appears due to the electric field effect at approximately kT=0.025 eV; this is associated with a significant shift in the spectrum corresponding to the ellipsoidal quantum dot.

**Figure 8:**
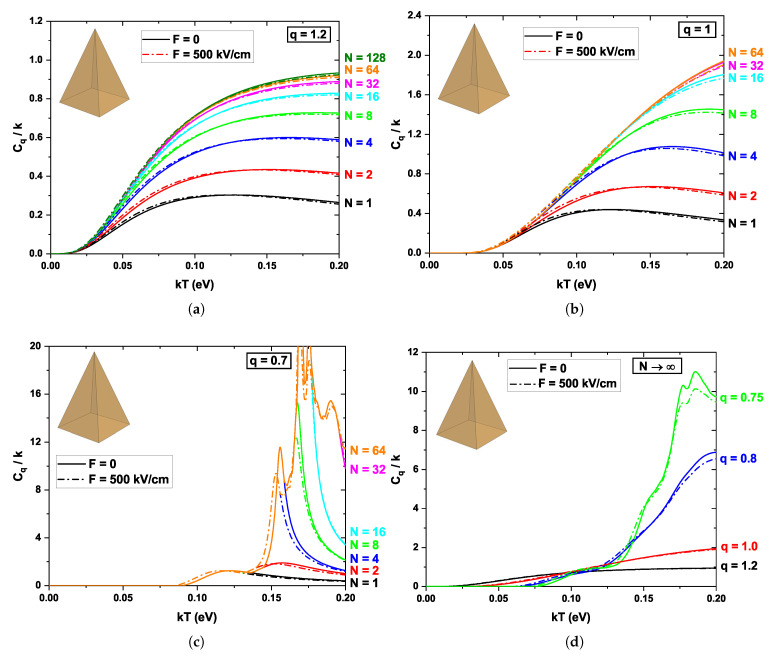
Variations of the normalized specific heat $C_{q}/k$ for the pyramidal quantum dot as a function of the parameter kT for different values of the non-extensivity parameter *q*. The solid and dashed lines represent F=0 and F=500 kV/cm, respectively. (**a**) $C_{q}/k$ as a function of kT for q=1.2. The colors of each line represent an increase in the number of states considered (see the *N* column on the right side of the panel). (**b**) Same as in panel (**a**) for q=1. (**c**) Same as in panel (**a**) for q=0.7. (**d**) $C_{q}/k$ as a function of kT for a very large number of states N→∞ and for different values of the parameter *q*.

Figure 8 shows the normalized specific heat, $C_{q}/k$, as a function of temperature for the pyramidal quantum dot, considering different numbers of states *N* and various values of the nonextensivity parameter *q*, including the effect of the external electric field. In Figure 8a and Figure 8b, corresponding to the cases q=1.2 and q=1, respectively, a smooth behavior is observed that tends to converge as the number of considered states increases. Unlike the previously analyzed quantum dots, the specific heat in this case shifts toward higher values of kT before becoming nonzero. This effect arises from the upward shift of the ground-state energy, which requires a higher thermal excitation energy compared to the previous quantum dot geometries (see Figure 4a). In Figure 8c, convergence in the specific heat is reached with a smaller number of states (N=64 in this case, compared to N=256 for the ellipsoidal QD, for example), and sharper peaks are observed in $C_{q}/k$. This is a direct consequence of the strong quantum confinement in the pyramidal structure, in which the energy levels are well-defined and remain separated up to relatively high energies. When the thermal energy is sufficient to populate a new level, a sudden increase in specific heat capacity occurs. The rate at which the specific heat convergence is achieved depends on the geometric confinement and symmetry of the QDs. Geometries with stronger confinement and lower degeneracy, such as the pyramidal quantum dot, require fewer energy levels to reach convergence, whereas geometries with higher symmetry, such as the cubic and cylindrical cases, demand a larger number of states due to increased level degeneracy and denser spectra. For all geometries and electric field configurations considered, the retained eigenstates span an energy range well above the thermal energy scale kT corresponding to the maximum temperature explored. As a result, all thermally accessible states that contribute significantly to the partition function are included, ensuring that the computed internal energy and specific heat are fully converged and unaffected by spectral truncation. This analysis confirms that the reported geometry-dependent thermal features are intrinsic to the quantum confinement and nonextensive statistics, rather than artifacts arising from an insufficient number of energy levels.

Finally, in Figure 8d, within the same range kT, the system response shifts to higher temperatures, reflecting the stronger confinement of the pyramidal dot compared to the other geometries. It is also noted that the effect of the electric field applied along the *x*-direction is minimal in all cases (compare solid and dashed lines). This occurs because electrons are strongly confined along that direction and the electric field only produces a slight shift in the wave function (see Figure 4b). Within the present framework, thermal stability can be directly assessed through the behavior of the internal energy and the specific heat as functions of temperature. Smooth, saturation-like behavior of the specific heat (particularly in the extensive and superextensive regimes (q≥1)) indicates a stable redistribution of carriers among energy levels under thermal excitation. In contrast, the appearance of sharp peaks in subextensive regimes (q<1) reflects a strong sensitivity to thermal fluctuations associated with the discrete nature of the spectrum. Therefore, the proposed approach provides a consistent way to analyze both the spectral range and the thermal robustness of the system.

A comparative analysis of the four geometries reveals that the QD shape plays a decisive role in determining both the energy spectrum and the resulting thermal response. The cubic and cylindrical QDs, which possess higher symmetry, exhibit a denser energy spectrum and greater sensitivity to the external electric field, resulting in more pronounced shifts in position and degeneracy and smoother variations in specific heat with respect to the external electric field. In contrast, the elipsoidal QD shows a denser energy spectrum and a stronger sensitivity to the external electric field, leading to more pronounced shifts in the position of the specific-heat peaks. The pyramidal QD, characterized by the strongest confinement and lowest symmetry, displays higher ground-state energies, sharper peaks in the specific heat, and faster convergence with respect to the number of included states. These differences highlight how geometric confinement directly controls the redistribution of energy levels and the emergence of Schottky-like anomalies under both extensive and nonextensive statistical regimes.

The robustness of the present results has been tested against variations in key parameters such as the nonextensivity index *q*, the strength of the external electric field, and the temperature. While these parameters quantitatively modify the occupation probabilities and shift the characteristic features of the specific heat, the qualitative trends associated with geometric confinement and symmetry remain unchanged. This demonstrates that the reported behaviors are intrinsic to the interplay between geometry and nonextensive statistics rather than numerical artifacts.

**Figure 9:**
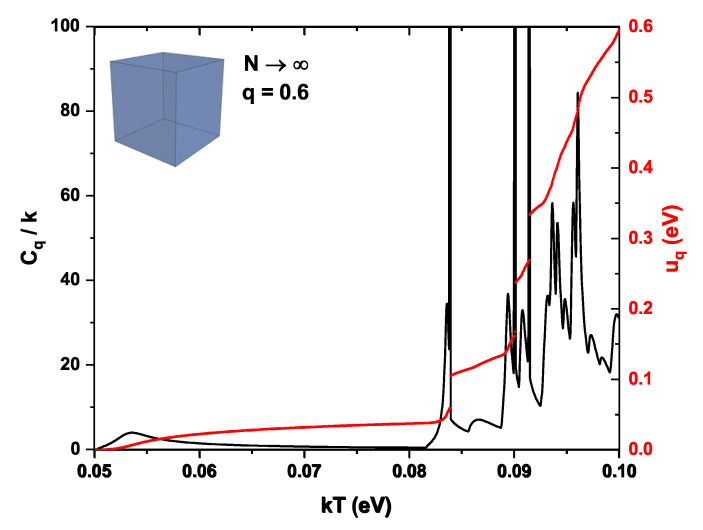
$C_{q}/k$ as a function of kT for a very large number of states N→∞, for the parameter q=0.6 in the cubic system (Left axis, black curve). The red curve, represented on the right axis, corresponds to the internal energy $u_{q}$.

As the nonextensivity parameter *q* decreases, the statistical distribution places greater weight on lower-energy states. However, this also implies that when the thermal energy increases, the energy levels remain well defined and, once the available energy becomes sufficient to reach them, an abrupt rise in the internal energy occurs. This behavior is illustrated in Figure 9 (red curve), which shows the internal energy as a function of kT for q=0.6 and the cubic QD.

The abrupt jumps are located approximately at kT=0.083 eV, kT=0.09 eV, and kT=0.092 eV. For these values, the specific heat tends to diverge, showing a Dirac delta–like behavior; however, from a physical perspective, it should be emphasized that in real QDs such sharp features would be smoothed by broadening mechanisms such as finite lifetime effects, electron–phonon interactions, and structural disorder. Consequently, the reported delta-like structures should be interpreted as extremely sharp peaks rather than true mathematical divergences.

It is important to mention that the appearance of deviations from the Boltzmann–Gibbs formalism in nanoscopic systems is commonly associated with the absence of a strict thermodynamic limit, strong spatial confinement, and the discrete nature of the energy spectrum. In GaAs QDs, these factors can give rise to effective correlations among accessible states, enhanced sensitivity to environmental coupling, and fluctuations in local thermodynamic quantities. In this context, nonextensive statistical mechanics provides an effective framework for accounting for such effects in a unified manner. In this development, the nonextensivity index *q* is not interpreted as a fundamental constant but rather as an emergent parameter that encapsulates the combined influence of confinement-induced correlations, finite-size effects, and possible deviations from equilibrium. From this perspective, *q* plays a role analogous to an effective parameter characterizing how the occupation of energy levels deviates from the standard extensive scenario, without requiring a specific microscopic mechanism.

## 4. Conclusions

In this work, the thermal response (normalized specific heat $C_{q}/k$) of GaAs QDs with cubic, cylindrical, ellipsoidal, and pyramidal geometries has been systematically analyzed within the framework of Tsallis nonextensive statistics. By combining geometric confinement, external electric fields, and generalized statistical effects, we have shown that the QD shape strongly influences the energy levels and, consequently, the specific heat behavior.

For superextensive regimes (q>1), the specific heat exhibits smooth, saturation-like behavior, whereas for subextensive regimes (q<1), an effective cutoff in the accessible energy states leads to sharp peaks and a discrete thermal response. The application of an external electric field induces geometry-dependent shifts in the specific heat, which are particularly pronounced in the ellipsoidal QD and minimal in the pyramidal case due to strong localization.

Among the results, Schottky-like anomalies induced by the external electric field were observed and can be attributed to the field-induced energy shift. In pyramidal QD, the effect of the field along the *x*-direction is minimal due to the strong localization of the wave function. In contrast, in an ellipsoidal QD, the shift of the ground state is highly significant.

The above results reveal localized peaks, shifts in $C_{q}$ under an external field, and a functional dependence on the non-extensivity parameter *q*. These results demonstrate that the combined use of geometry and nonextensive statistics provides a robust framework for tailoring and interpreting thermal properties in nanoscopic quantum systems.

## Figures and Tables

**Table 1:** Parameters used in this work.

Parameter	Value
$m^{*}$	0.067 $m_{0}$
$m_{0}$	9.109 × $10^{-31}$ kg
*k*	1.381 × $10^{-23}$ J/K
*ℏ*	6.582 × $10^{-16}$ eV s

**Table 2:** Number of states for optical transitions.

System	F=0			F=500 kV/cm		
	Energy Range (eV)			Energy Range (eV)		
	0.0–0.4	0.4–0.8	0.8–1.2	0.0–0.4	0.4–0.8	0.8–1.2
Cube	2	4	4	3	7	6
Cylinder	3	7	8	4	9	10
Ellipsoid	3	7	9	3	7	11
Pyramid	1	2	6	1	3	7

## Data Availability

The original contributions presented in this study are included in the article. Further inquiries can be directed to the corresponding author.
